# Genome Assembly of *Chryseobacterium polytrichastri* ERMR1:04, a Psychrotolerant Bacterium with Cold Active Proteases, Isolated from East Rathong Glacier in India

**DOI:** 10.1128/genomeA.01305-15

**Published:** 2015-11-05

**Authors:** Rakshak Kumar, Dharam Singh, Mohit Kumar Swarnkar, Anil Kumar Singh, Sanjay Kumar

**Affiliations:** Department of Biotechnology, CSIR, Institute of Himalayan Bioresource Technology, Palampur, Himachal Pradesh, India

## Abstract

We report here the genome assembly of a psychrotolerant bacterium, *Chryseobacterium polytrichastri* ERMR1:04, which secretes cold-active proteases. The bacterium was isolated from a pristine location, the East Rathong Glacier in the Sikkim Himalaya. The 5.53-Mb genome provides insight into the cold-active industrial enzyme and adaptation in the cold environment.

## GENOME ANNOUNCEMENT

The East Rathong Glacier in the Sikkim Himalaya has been explored for changes in its glacier volume and geologic importance (1–3), but reports on microbes there are lacking. The cold environment of glaciers may harbor a diverse bacterial community with a potential source of industrially important cold-active enzymes (4). Realizing this importance, we isolated cold-adapted bacteria at 5°C from an East Rathong Glacier sample and found few potentially prospective protease-secreting bacteria after screening, using a plate assay, as described earlier (5). Amongst them, ERMR1:04 is a yellow-pigmented, Gram-negative, aerobic, and nonmotile bacterium that was isolated from a soil sample collected from the moraines (27°33′049′′N 88°07′533′′E; 4,613 meters above sea level [masl]). Using its 16S rRNA gene sequence (1,506 bp), phylogenetically, it was found to branch with the type strain of *Chryseobacterium polytrichastri* YG4-6 with 98.92% sequence similarity, confirming that the strain belongs to species *C. polytrichastri* (6). The type strain (YG4-6) was reported from a glacier in China; however, its genome data are not available (7). Therefore, to elucidate genomic insights into industrial enzymes and its cold adaptation, the strain ERMR1:04 was chosen for whole-genome sequencing.

Genomic DNA from strain ERMR1:04 was isolated using a standard genomic DNA isolation method. The quality and quantity of extracted genomic DNA were determined by a Qubit 2.0 fluorometer (Invitrogen, USA). Genomic DNA was sheared using Covaris g-Tubes to construct a library with a 10-kb DNA insert size using the PacBio SMRTbell template preparation kit version 1.0, as described earlier (8). The prepared SMRTbell library was sequenced using the PacBio RS II system on two SMRT cells using P6 polymerase-C4 sequencing chemistry with a 240-min movie. Two SMRT cells produced 1,832,764,456 nucleotide bases generated from 118,266 reads (*N*_50_ size, 27,831 bp; mean read length, 15,496 bp). The filtered subreads were assembled *de novo* using CLC Genomics Workbench version 8.0.3 (Qiagen Aarhus A/S). The strain ERMR1:04 genome was assembled in 10 contigs, for a total of 5,532,908 bp in size with 34.07% G+C content (longest contig length, 1,673,155; *N*_50_ contig length, 1,207,894; mean contig length, 553,291).

The protein-coding sequences (CDSs) were predicted by the Prokaryotic Genome Annotation Pipeline (PGAP) version 2.10 (NCBI, 2013), which predicted 4,938 genes, 4,524 CDSs, 24 rRNAs (5S, 16S, 23S), 75 tRNAs, 1 noncoding RNA (ncRNA), and 314 pseudogenes. Functional annotation was performed using the Rapid Annotations using Subsystems Technology (RAST) server (9), which predicted 5,176 coding sequences and 106 RNAs. These predicted genes were functionally assigned to 357 RAST subsystem categories. The genomic analysis identified genes responsible for industrially important enzymes, such as protease, catalase, permease, esterase/lipase, chitinase, alcohol dehydrogenase, glycosyltransferase, and alkaline phosphatase. Our experimental data of cold-active protease is well corroborated with the presence of various genes encoding important proteases, such as Clp protease, HtrA protease, and metalloproteases in the genome. Cold-shock proteins (CspA and CspG), which aid in the survival of bacteria in harsh environments, were also found.

This is the first report of genomic data of the species *C. polytrichastri*, and it will aid in comparative genomic studies for understanding the evolutionary mechanism of cold adaptation and cold-active industrial enzymes.

### Nucleotide sequence accession numbers.

This whole-genome shotgun project of *C. polytrichastri* ERMR1:04 has been deposited at DDBJ/EMBL/GenBank under the accession no. LIRF00000000. The version described in this paper is version LIRF01000000**.**
